# Genome Sequences of Polyomaviruses from the Wild-Living Red Colobus (*Piliocolobus badius*) and Western Chimpanzee (*Pan troglodytes verus*)

**DOI:** 10.1128/genomeA.01101-16

**Published:** 2016-10-13

**Authors:** Nicole Ben Salem, Fabian H. Leendertz, Bernhard Ehlers

**Affiliations:** a Division 12, Measles, Mumps, Rubella and Viruses Affecting Immunocompromised Patients, Robert Koch Institute, Berlin, Germany; b P3, Epidemiology of Highly Pathogenic Microorganisms, Robert Koch Institute, Berlin, Germany

## Abstract

We identified with PCR and sequencing the full genomes of the recently discovered *Pan troglodytes verus* polyomavirus 8 and *Piliocolobus badius* polyomavirus 2 in a western chimpanzee and a western red colobus free-ranging in Taï National Park of Côte d’Ivoire.

## GENOME ANNOUNCEMENT

Polyomaviruses have been discovered in a wide range of nonhuman primates; however, a considerable number have been either identified only in captive individuals or discovered based only on short genome fragments, such as *Pan troglodytes verus* polyomavirus 8 (PtrovPyV8) and *Piliocolobus badius* polyomavirus 2 (PbadPyV2). PtrovPyV8 was discovered in 2016 in necropsy samples of the uro- and gastrointestinal tract of a captive chimpanzee individual (name: Régina) and housed at the Biomedical Primate Research Centre in The Netherlands (RefSeq accession no. NC_028635) (1), and PbadPyV2 was discovered in 2013 in a spleen necropsy sample of a western red colobus free-ranging in Taï National Park of Côte d’Ivoire (GenBank accession no. JX159996) (2). While the PtrovPyV8 genome had been amplified and sequenced to completion, only 203 bp of the VP1 gene of PbadPyV2 had been reported.

Here, we report the full genomes of PtrovPyV8, isolated from the spleen (isolate #4699) of a free-ranging chimpanzee (name: Orest), and PbadPyV2, isolated from an axillary lymph node (isolate #5947) from a red colobus. Both individuals were found dead in Taï National Park and necropsied on-site in 2005. Amplification and sequencing was done essentially as described before (3). The PtrovPyV8 and PbadPyV2 viruses have genomes of 5,164 bp and 5,148 bp, respectively, with an organization of coding sequences (CDSs) typical for polyomaviruses, including an agnoprotein CDS (PtrovPyV8). Compared to the PtrovPyV8-Regina genome, the PtrovPyV8-Orest genome displays a nonsynonymous nucleotide (nt) exchange in the large T antigen (LTAg) CDS and an nt insertion in the intergenic region between VP1 and the LTAg CDS. Using the MAFFT module in Geneious version 9.1.3 software, both PtrovPyV8 genomes display closest pairwise nucleotide identity (89%) to the genome of *Pan troglodytes troglodytes* polyomavirus 1 (5,159 bp; accession no. NC_027532), recently described in a wild-living central chimpanzee (4), and 80% identity to the most closely related human polyomavirus (BK virus; RefSeq accession no. NC_001538). The PbadPyV2 genome reveals closest identity (90%) to the genome of *Piliocolobus rufomitratus* polyomavirus 1 (5,140 bp; RefSeq accession no. NC_019850), identified earlier in a wild-living eastern red colobus (2), and 69% to the human New Jersey polyomavirus (RefSeq accession no. NC_024118). This close genetic relatedness of panine as well as colobine polyomaviruses detected in their natural, free-ranging hosts confirms that cospeciation is a major force of polyomavirus evolution.

### Accession number(s).

The complete genomes PtrovPyV8 isolate 4699 and PbadPyV2 isolate 5947 have been deposited in GenBank under the accession numbers KU865500 and KX509984.
